# Änderungen des Arbeitsalltags und Moral Distress in der psychiatrischen Versorgung während der COVID-19-Pandemie: Eine Umfrage unter stationär tätigen Ärzt*innen in Deutschland

**DOI:** 10.1007/s00115-023-01499-z

**Published:** 2023-07-05

**Authors:** Jeanne Guinaudeau, Paul Christian Baier, Katja Kühlmeyer, Christoph Borzikowsky, Laura Terheyden, Victoria Dorothea Witt, Annette Rogge

**Affiliations:** 1 Zentrum für Integrative Psychiatrie, Universitätsklinikum Schleswig-Holstein, Niemannsweg 147, 24105 Kiel, Deutschland; 2 Institut für Ethik, Geschichte und Theorie der Medizin, Ludwig-Maximilians-Universität München, München, Deutschland; 3 Institut für Medizinische Informatik und Statistik, Christian-Albrechts-Universität zu Kiel, Universitätsklinikum Schleswig-Holstein, Campus Kiel, Kiel, Deutschland; 4 Klinik für Neurologie, Nordseeklinik Helgoland, Helgoland, Deutschland; 5 Klinik für Neurologie, Universitätsklinikum Schleswig-Holstein, Kiel, Deutschland; 6 Sozialpsychiatrie und Gesundheitsförderung Kreis Segeberg, Bad Segeberg, Deutschland

**Keywords:** Moralische Herausforderungen, Klinische Ethik, Public-health-Ethik, Psychiatrie, Allokation, Moral challenges, Clinical ethics, Public health ethics, Psychiatry, Allocation

## Abstract

**Hintergrund:**

Während der COVID-19-Pandemie ist es zu besonderen moralischen Herausforderungen im Gesundheitswesen gekommen. Eine psychische Reaktion auf moralische Herausforderungen wird als Moral Distress (MD) bezeichnet.

**Ziel der Arbeit:**

Identifikation von Ursachen für MD in der stationären psychiatrischen Versorgung im Kontext der COVID-19-Pandemie in Deutschland.

**Material und Methoden:**

Es wurde eine Umfrage mit einem selbstadministrierten nichtvalidierten Onlinefragebogen als Querschnittserhebung durchgeführt, in dem 26 Items zum Erleben von MD untersucht sowie offene Fragen zum Umgang mit der Pandemie und Auswirkungen auf den Arbeitsalltag qualitativ ausgewertet wurden. Ärzt*innen, die in der stationären psychiatrischen Versorgung während der COVID-19-Pandemie in Deutschland tätig waren, wurden anonym mit einer Gelegenheitsstichprobe befragt. Die Erhebung fand vom 17.11.2020 bis 06.05.2021 statt.

**Ergebnisse:**

Es wurden 141 Teilnehmer*innen eingeschlossen. Sie gaben vielfältige pandemiebedingte Veränderungen des Arbeitsalltages an, die teilweise in MD resultierten.

**Diskussion:**

Moral Distress stellt für Ärzt*innen in der stationären psychiatrischen Versorgung unter Pandemiebedingungen (und darüber hinaus) einen potenziellen Belastungsfaktor dar, der weitere Forschung und einen angemessenen Umgang erfordert. Es ergeben sich sowohl Implikationen für Entscheidungstragende in Krisenstäben als auch ein Bedarf für Unterstützungsangebote z. B. durch Dienste zur klinischen Ethikberatung.

**Zusatzmaterial online:**

Die Onlineversion dieses Beitrags (10.1007/s00115-023-01499-z) enthält weitere Tabellen.

## Abstract (en)

**Background:**

During the COVID-19 pandemic a number of ethical challenges have arisen in the healthcare system. A psychological response to moral challenges is termed moral distress (MD).

**Objective:**

Identification of causes of MD in inpatient psychiatric care in the context of the COVID-19 pandemic in Germany.

**Material and methods:**

A survey was conducted using a self-administered non-validated online questionnaire as part of a cross-sectional study, in which 26 items about the experience of MD were examined and open questions about the handling of the pandemic and its effects on everyday work were posed. Physicians who worked in inpatient psychiatric care during the COVID-19 pandemic in Germany were surveyed anonymously with a convenience sample. The data acquisition took place between 17 November 2020 and 6 May 2021.

**Results:**

A total of 141 participants were included. They indicated multiple pandemic-related changes in their daily work partly resulting in MD.

**Conclusion:**

MD is a neglected potential burden of inpatient psychiatric care under pandemic conditions (and beyond), which requires further research and an adequate handling. These results include implications for decision makers in crisis teams as well as a need for support services such as clinical ethics consultation services.

Moral Distress (MD) bezeichnet eine psychische Reaktion auf moralische Herausforderungen, z. B. durch moralische Konflikte, moralische Unsicherheit oder Barrieren bei der Umsetzung moralischen Handelns. Eine wissenschaftliche Beschäftigung mit MD bei Gesundheitsfachkräften findet unter anderem in der klinischen Ethik statt. Unter der Annahme, dass eine Public-health-Krise spezifische Herausforderungen mit sich bringt, erfolgt hier eine Untersuchung von MD bei Psychiater*innen in der stationären Versorgung während der COVID-19-Pandemie.

## Hintergrund und Fragestellung

Ein Faktor, der die psychische Gesundheit und die moralische Integrität von Gesundheitspersonal gefährden kann, ist Moral Distress (MD). Nach Fouriers weit gefasster Definition bezeichnet MD eine psychische Reaktion auf moralisch herausfordernde Situationen wie das Erleben von moralischer Unsicherheit, moralischen Konflikten bzw. Dilemmata oder von Barrieren bei der Umsetzung moralischen Handelns [1]. Corley betont insbesondere institutionelle Handlungseinschränkungen als Auslöser von MD und hebt damit die institutionelle Verantwortung hervor, Bedingungen für ethisch begründetes Handeln zu schaffen und moralisch gebotenes Handeln nicht zu behindern [2]. Neuere Arbeiten weisen dabei besonders auf die Verantwortung von Führungskräften hin, die selbst von MD betroffen sein können [3].

Die COVID-19-Pandemie hat für Akteur*innen im Gesundheitswesen zum Teil neue Herausforderungen im beruflichen Handeln geschaffen, zum Teil bekannte Herausforderungen verstärkt [4–6]. Pandemiespezifische moralische Herausforderungen begründeten sich etwa darin, dass der Fokus auf das individuelle Patient*innenwohl gegen den Schutz der öffentlichen Gesundheit (wie Infektionsschutz) abgewogen werden muss, was vor der Pandemie im psychiatrischen Bereich nur in Ausnahmefällen, z. B. im Rahmen des Schutzes vor Patient*innen mit fremdgefährdendem Verhalten, notwendig war [7].

Moral Distress wurde im psychiatrischen Kontext auch schon vor der COVID-19-Pandemie identifiziert. Für Pflegende waren Auslöser z. B. Fälle von Zwangsbehandlung und vermeidbaren Zwangsmaßnahmen, eine Zunahme von Patient*innen mit schweren Erkrankungen, wenig personelle Ressourcen und Gewalt am Arbeitsplatz [8]. Gespräche mit Kolleg*innen können Betroffenen helfen, MD zu begegnen [9]. Darüber hinaus gibt es Überlegungen durch eine Kultur ethischer Praxis gezielt moralische (Team‑)Resilienz aufzubauen [9].

Es gibt eine Vielzahl an Studien, die sich mit MD während der COVID-19-Pandemie beschäftigen [10], wobei beispielsweise fehlende Schutzausrüstung, Beeinträchtigung der Patient*innenversorgung durch Hygienemaßnahmen sowie Verknappung von Zeit und Personal als Auslöser thematisiert wurden [11, 12]. Guter Kommunikationsstil seitens der Führungspositionen wurde dabei als ein Faktor herausgearbeitet, der pandemiebedingten MD reduzieren kann [11]. Zudem wurden klare Handlungsanweisungen zur Priorisierung von Gesundheitsressourcen, aber auch entlastende Gespräche mit Familie und Freund*innen als hilfreich empfunden [13].

Bisher konnten wir keine Studien identifizieren, in der MD in der stationären psychiatrischen Versorgung in der COVID-19-Pandemie untersucht wurde. Besondere Herausforderungen bestehen in einer Pandemie für die psychiatrische Patient*innenversorgung unter anderem durch Auswirkungen von freiheitseinschränkenden Infektionsschutzmaßnahmen auf ohnehin schon stark eingeschränkte, gesetzlich untergebrachte Personen. Darüber hinaus werden Nutzen‑/Schadenserwägungen bei Entscheidungen über Zwangs- und Isolationsmaßnahmen durch weitere potenzielle Schadenspotenziale wie z. B. Ansteckungsgefahren von Patient*innen verschärft. Hinzu kommen Erschwernisse bei der Erfüllung der Notwendigkeit richterlicher Anhörungen zur Entscheidung über Unterbringung und Zwangsbehandlung. Außerdem ist die Auswirkung pandemiebedingter Stressoren auf die psychische Gesundheit des Personals zu bedenken [5, 14, 15].

Die vorliegende Arbeit hat das Ziel, das Ausmaß von MD im Kontext der stationären psychiatrischen Versorgung während der COVID-19-Pandemie zu untersuchen, die wichtigsten Ursachen für MD zu identifizieren und pandemiebedingte Änderungen des Arbeitsalltages zu erfassen. Eine solche Untersuchung kann ethische Herausforderungen identifizieren und Unterstützungsbedarfe aufzeigen, die im Rahmen organisational verankerter Dienste, wie z. B. der klinischen Ethikberatung, aufgegriffen werden können [16, 17]. Über eine Pandemie hinaus kann eine solche Untersuchung für andere Gesundheitssystemkrisen relevant sein, die zu einem Wandel moralischer Auffassungen führen können.

## Studiendesign und Untersuchungsmethoden

Die Beschreibung des Studiendesigns und der Forschungsmethoden erfolgt nach der Checkliste für die Berichtlegung von Internet-E-Surveys (CHERRIES; [18]).

Die Befragung fand im Rahmen einer Querschnittserhebung anhand eines selbstadministrierten Onlinefragebogens statt. Ärzt*innen, die in der stationären psychiatrischen Versorgung (Unikliniken, Fachkliniken und Fachabteilungen) während der COVID-19-Pandemie in Deutschland tätig waren, wurden anonym mit einer Gelegenheitsstichprobe befragt. Die zuständige Ethikkommission bescheinigte die Unbedenklichkeit der Untersuchung (D 621/20). Von allen Teilnehmenden liegt eine Einverständniserklärung zur Datenerhebung, Verarbeitung und Speicherung entsprechend den gesetzlichen und ethischen Vorgaben zur informierten Einwilligung vor.

### Entwicklung des Erhebungsinstruments und Pretest

Zum Zeitpunkt der Untersuchung stand kein validiertes Erhebungsinstrument für den Studienzweck zur Verfügung. Die Entwicklung des Fragebogens war angelehnt an die Moral Distress Scale-Revised [19]. Zur Fragebogenentwicklung wurden offene Einzelinterviews mit vier psychiatrischen Assistenzärzt*innen geführt. Mögliche Situationen, die MD verursachen könnten, wurden identifiziert und unter Einbezug der Besonderheiten der Pandemie in einer interdisziplinären Arbeitsgruppe (Psychiatrie, Klinische Ethik und Psychologie) diskutiert. In der Pilotierungsphase wurde der vorläufige Fragebogen durch fünf Psychiater*innen getestet. Der endgültige Fragebogen bestand aus 26 Items zum Themengebiet MD und 8 Fragen zum allgemeinen Umgang mit der Pandemie und offene Fragen zu möglichen pandemiebedingten Änderungen des Arbeitsalltages. Die geschlossenen Fragen konnten auf einer Likert-Skala beurteilt werden.

### Auswahl und Rekrutierung von Studienteilnehmer*innen

Der Erhebungszeitraum war vom 17.11.2020 zum 06.05.2021. Der Link zur anonymen Teilnahme wurde nach Internetrecherche stationärer psychiatrischer Kliniken an 380 Chefärzt*innen zur Weitergabe versandt. Die Datenerhebung und -speicherung erfolgte automatisch mit dem Online-Tool SosciSurvey (SoSci Survey GmbH, München, Deutschland). Die Teilnahme konnte ohne Transaktionsnummer (TAN) erfolgen, sodass theoretisch ein mehrfaches Ausfüllen der gleichen Person nicht ausgeschlossen werden kann.

### Datenanalyse

Es wurden nur Datensätze in die Analyse eingeschlossen, bei denen die Studienteilnehmer*innen während der COVID-19-Pandemie im stationären Kontext gearbeitet haben (*n* = 141). Zur Auswertung wurden Median und 1./3. Quartil sowie der Modus (Modalwert) errechnet. Für die Items zu MD wurde mit einem Teil der Stichprobe Cronbachʼs α als Maß der internen Konsistenz genutzt.

Neben dem geschlossenem Fragenteil ergaben zwei Freitextantworten umfassende Daten, die mithilfe einer zusammenfassenden qualitativen Inhaltsanalyse nach Mayring ausgewertet wurden [20].

## Ergebnisse

### Stichprobe

Es wurden 228 (100 %) Datensätze gespeichert, in die Auswertung gingen 141 (61,8 %) Studienteilnehmer*innen ein. Ausgeschlossen wurden Datensätze, bei denen keine Angabe zum Einsatzort (stationär/ambulant) oder die Angabe einer rein ambulanten Tätigkeit in der Pandemie gemacht wurde.

### Quantitative Ergebnisse

#### Änderung des Arbeitsalltags

Insgesamt gaben 136 Teilnehmende (96,5 %) an, dass ihr Arbeitsalltag eine Änderung durch die COVID-19-Pandemie erfahren habe. Davon stimmten 105 (77,2 %) Teilnehmende der Aussage zu, dass die Veränderung eine Verschlechterung der Patient*innenversorgung bedeutete. Insgesamt 122 Teilnehmende (86,5 %) hatten bereits Kontakt mit Patient*innen mit (Verdacht auf) COVID-19. 133 Teilnehmende (94,3 %) hatten Kontakt zu Patient*innen, die aufgrund der Pandemie psychische Betreuung benötigten.

#### Auslöser von MD

Die Antworten zu den einzelnen Items sind getrennt für Frequenz und für die erlebte Stressintensität in Tab. 1 dargestellt.

**Table 1:** 

	Frage	Frequenz						Stressintensität					
		n	Q1	Md	Q3	Mo	n/Mo	n	Q1	Md	Q3	Mo	n/Mo
1	Die ärztliche Diagnostik, Beratung und Behandlung konnte nicht am medizinischen Bedarf orientiert durchgeführt werden	140	2	3	4	4	49	140	2	3	4	4	50
2	Die ärztliche Diagnostik, Beratung und Behandlung konnte nicht an den Wünschen der Patient*innen orientiert durchgeführt werden	140	3	4	5	4	50	140	2	3	4	3	46
3	Die selbstbestimmte Gestaltung meines Arbeitsalltags als Ärzt*in war nur eingeschränkt möglich	139	2	3	4	4	42	139	2	3	4	2; 4	38
4	Meine übliche Arbeitsroutine wurde durch Verfahrensanweisungen, Schutzkleidung und/oder Umorganisation der Arbeitsabläufe (z. B. verkürzte Kontaktzeit mit Patient*innen, weniger Patient*innentermine pro Tag o. Ä.) eingeschränkt	140	3	4	5	5	48	140	2	3	4	4	38
5	Die Begleitung von Patient*innen durch Angehörige war erschwert	140	4	5	5	5	95	140	2,25	3	4	3	43
6	Durch die Einschränkungen von Angehörigenbesuchen bei Patient*innen entfiel eine Entlastung in der Patient*innenbetreuung	140	3	4	5	4	38	140	2	3	4	2	39
7	Durch die fehlende Evidenz von Empfehlungen (z. B. Testsicherheit, Schutzmaßnahmen, wer ist Risikogruppe?) entstand für mich Unsicherheit im Umgang mit Verdachtsfällen von COVID-19	140	2	3	4	2; 3	36	140	2	3	4	3; 5	33
8	Die Einschätzung, welches ärztliche Handeln ethisch/moralisch richtig war, ist mir nicht leicht gefallen	140	2	3	3	2	41	140	2	3	4	2	41
9	Die Handlungsempfehlungen verschiedener Fachgesellschaften bzw. der Regierung haben sich gegenseitig widersprochen	140	2	3	4	3	39	140	2	3	4	2	36
10	Das Tragen eines Mund-Nasen-Schutzes erschwerte die Kommunikation mit Patient*innen und Angehörigen oder Kolleg*innen und Vorgesetzten	140	4	4	5	5	62	140	2	3	4	3	39
11	Durch fehlendes Material (z. B. zum Infektionsschutz) kam es zu Engpässen in der Einhaltung der Vorgaben	140	1	2	4	2	40	140	1	2	4	2	42
12	Durch fehlendes Personal kam es zu Engpässen in der Einhaltung der Vorgaben	140	2	3	4	2; 4	33	140	2	3	4	5	34
13	Durch Mangel an administrativen Vorgaben konnte ich der Patient*innenversorgung nicht angemessen gerecht werden	139	1	2	3	2	47	139	1	2	3	1	45
14	Es hat bei untergebrachten Patient*innen Situationen gegeben, bei denen ich zum Infektionsschutz Zwangsmaßnahmen durchführen musste (z. B. Zimmerisolierung)	137	1	3	4	1	39	136	1	3	4	1	39
15	Es hat bei freiwillig aufgenommenen Patient*innen Situationen gegeben, bei denen ich zum Infektionsschutz Zwangsmaßnahmen durchführen musste (z. B. Zimmerisolierung)	138	1	3	4	1	41	136	1	2	4	1	41
16	Es ist zu Situationen gekommen, in denen aufgrund von Noncompliance der Infektionsschutzmaßnahmen eine disziplinarische Entlassung erfolgte	139	1	2	3	1	47	137	1	2	3	1	50
17	Es ist zu Situationen gekommen, in denen aufgrund von Noncompliance der Infektionsschutzmaßnahmen die Unterbringung als Zwangsmaßnahme richterlich beantragt werden musste	138	1	1	2	1	80	136	1	2	3	1	67
18	Eine Fixierung oder medikamentöse Ruhigstellung erfolgte länger als ich es vor der Pandemie für nötig erachtet habe	139	1	1	1	1	111	137	1	1	2	1	93
19	Die stationäre Behandlungsdauer war nicht ausreichend, um das Therapieziel zu erreichen	139	1	2	3	1	42	138	1	2	3	1	40
20	Ich musste Patient*innen länger als vorhergesehen auf der Station behalten, was Patient*innen, Angehörige oder Personal belastet hat	139	1	3	3	1	39	137	1	2	4	1	37
21	Chronisch kranke Patient*innen wurden im Hinblick auf das Therapieziel Heilung oder Rehabilitation behandelt, obwohl diese Ziele offensichtlich nicht mehr zu erreichen waren	138	1	2	2	1	68	137	1	2	3	1	65
22	Ein palliatives Therapiekonzept wurde bei Patient*innen unverhältnismäßig früh diskutiert	137	1	1	1	1	120	135	1	1	2	1	100
23	Patient*innen sind zu wichtigen Untersuchungen/Behandlungsterminen nicht erschienen	139	1	2	3	2	38	139	1	2	3	1; 2	47
24	Bei Patient*innen mit chronischen Erkrankungen musste ich meine Versorgung unterbrechen	138	1	2	3	1	63	136	1	2	3	1	58
25	Mir sind Patient*innen begegnet, die z. B. aus Angst vor Ansteckung während eines Krankenhausaufenthaltes sehr lange gewartet haben, sich psychiatrische Hilfe zu suchen	139	2	3	4	2	33	139	2	2	3	2	43
26	Ich bin besorgt um Patient*innen, die sich gerade nicht in meiner Versorgung befinden (z. B. weil sie aufgrund allgemeiner Empfehlungen oder aus Angst vor einer Stigmatisierung zu Hause bleiben)	140	2	3	4	2	40	140	2	2,5	4	2	38

^a^Frequenz von 1 = nie bis 5 = sehr häufig und Stressintensität von 1 = gar nicht gestresst bis 5 = sehr gestresst. Dargestellt sind Rücklauf (*n*), Modus (*Mo*), 1. und 3. Quartil (*Q1, Q3*) sowie Median (*Md*)

Sowohl für Stressintensität (Cronbach’s α = 0,91) als auch für Frequenz (Cronbach’s α = 0,87) ergaben sich hohe interne Konsistenzen. Dies spricht für die Messung von zwei homogenen Konstrukten (Stressintensität und Frequenz).

Die fünf häufigsten Auslöser für MD standen im Zusammenhang mit der Einschränkung der Besuchsmöglichkeiten für Angehörige (5, 6), Erschwernissen in der Arbeit und Arbeitsorganisation durch pandemiebedingte Schutzmaßnahmen (10, 4) und einer fehlenden Patient*innenorientierung in der psychiatrischen Versorgung (2). Die fünf Auslöser, die den stärksten MD zur Folge hatten, waren zum Teil die gleichen (5, 10, 2). Hinzu kamen eine fehlende Bedarfsorientierung in der psychiatrischen Versorgung (1) und eine Unsicherheit im Umgang mit COVID-Verdachtsfällen als Auslöser für starken MD (7).

Am wenigsten bedeutsam (da weder häufig noch intensiv) wurden die Diskussion eines palliativen psychiatrischen Therapiekonzeptes (22), eine Fixierung oder medikamentöse Ruhigstellung der Patient*innen (18), eine Behandlung chronisch kranker Patient*innen auf das Therapieziel Heilung oder Rehabilitation, ohne dass es erreicht werden konnte (21), die Beantragung von Zwangsmaßnahmen aufgrund von Noncompliance mit Infektionsschutzmaßnahmen (17) oder die Unterbrechung der Versorgung chronisch Kranker (24) wahrgenommen.

### Qualitative Ergebnisse

#### Antworten auf offenen Fragen zu pandemiebedingten Änderungen im Arbeitsalltag

Die am häufigsten genannten Änderungen des Arbeitsalltages betrafen die psychiatrische Behandlung. Einerseits wurde ein Bemühen um eine gleichbleibende Behandlungsqualität und eine Therapieverbesserung bei niedrigeren Patient*innenzahlen beschrieben. Andererseits wurden vielfältige negative Änderungen thematisiert, insbesondere die Folgenden:

- ein geringeres Therapieangebot,
- mehr räumliche und *innere* Distanzierung zu den Patient*innen durch Hygieneabstände,
- die Zunahme somatischer Aspekte zuungunsten der psychotherapeutischen Betreuung,
- eine erschwerte Beurteilung der psychischen Verfassung,
- eine erschwerte Fokussierung der Patient*innen auf die Therapie und
- veränderte Erkrankungsverläufe oder Arten der Diagnosen (mehr Suizidalität, Anpassungsstörungen etc.)

Ebenfalls häufig wurden Änderungen in der Kommunikation beschrieben, wobei der Einfluss von Hygienemaßnahmen und deren negative Auswirkungen auf die Kommunikation (vorrangig das Fehlen der Mimik durch Tragen eines Mund-Nasen-Schutzes [MNS]) auch in den Freitextantworten eine wichtige Rolle spielten. Als weitere Aspekte mit Auswirkungen auf die Kommunikation wurden genannt:

- der eingeschränkte Austausch im Team,
- weniger Kommunikationsmöglichkeiten zwischen Therapeut*innen und Patient*innen durch Kontakteinschränkungen und
- das Ausprobieren neuer Kommunikationsmöglichkeiten (Videochat etc.)

Die Kommunikation über Infektionsschutzmaßnahmen mit den Patient*innen habe zusätzlich zeitliche Ressourcen beansprucht. Aber nicht nur Herausforderungen, auch positive Aspekte wie eine Zunahme der interdisziplinären Zusammenarbeit wurden berichtet.

Benannt wurde weiterhin eine veränderte Organisation des Arbeitsalltages, die mit einer Mehrbelastung des Personals, aber auch mit einer Verringerung der Qualität der Patient*innenversorgung einhergegangen sei. Dabei konnten gleichzeitig positive Aspekte wahrgenommen werden, wie mehr Ruhe und Rückzugsmöglichkeiten für einzelne Patient*innen durch weniger stationäre Aufnahmen.

Darüber hinaus wurden eine erschwerte soziale Interaktion im Kollegium, ein Arbeitsplatzwechsel durch Stationsschließungen, die Infektionsgefahr für Behandelnde und damit verbundene Konflikte bei der Behandlung infizierter Patient*innen, eine psychische Belastung der Behandelnden durch Angst und Unklarheiten benannt. Auch Personalausfall durch Quarantäne und Krankheit bzw. Einschränkungen in der Weiterbildung wurden erlebt.

Ein weiterer Aspekt war die Notwendigkeit zur Mittelallokation durch die pandemiebedingte Reduktion der stationären Patient*innenzahlen. Beispielsweise hätten Kliniken sich gezwungen gesehen, mehr Betten zu belegen, da Möglichkeiten der stationären Aufnahme durch pandemiebedingte Einschränkungen an anderen Kliniken nicht bestanden. Darüber hinaus nannten einige Teilnehmer*innen, dass Abwägungen zwischen individuellem Interesse der Patient*innen (in Bezug auf Infektionsschutz) und dem wirtschaftlichen Interesse der Kliniken notwendig gewesen seien. Von einigen Teilnehmer*innen wurde auch die Einschränkung der autonomen Lebensgestaltung von Patient*innen angegeben.

Auch in den Freitextantworten wurde erneut zur Sprache gebracht, dass die therapeutische Arbeit mit Angehörigen erschwert gewesen sei, da diese aufgrund von Infektionsschutzmaßnahmen weniger hätten einbezogen werden können.

Unsicherheiten im Umgang mit der Pandemie wurden vor allem in Bezug auf die Frage nach dem pandemiegerechten Vorgehen beschrieben. Von einigen Teilnehmer*innen wurde eine verzögerte Reaktion der Politik mit Maßnahmen der Pandemieeindämmung kritisiert. Vereinzelt wurden negative Gefühle in Bezug auf den Arbeitsalltag beschrieben. Eine Person thematisierte die Wahrnehmung einer Offenlegung von bereits bestehenden systemimmanenten Problemen durch die Pandemie.

## Diskussion

Dies ist eine der ersten Befragungen, in der MD bei psychiatrischen Fachkräften im deutschsprachigen Raum untersucht wurde. Die Ergebnisse zeigen, dass Psychiater*innen während der COVID-19-Pandemie von moralischem Stress betroffen waren. Die Mehrzahl der Teilnehmenden nahm pandemiebedingt eine Verschlechterung der psychiatrischen Versorgungsqualität wahr.

Moral Distress von Gesundheitspersonal während der COVID-19-Pandemie wurde auch in anderen Versorgungskontexten beschrieben [21–23]. In der Intensivmedizin wurden z. B. als Auslöser Ressourcenknappheit, Einsamkeit und Isolation der Patient*innen sowie Personalknappheit durch infizierte Mitarbeitende benannt [22]. Eine Beschreibung spezifischer Situationen, die MD in der Akutpsychiatrie auslösen können, findet sich bei Jansen et al. [8], allerdings unter Routinebedingungen und nicht in Bezug auf eine Pandemie.

Unsere Ergebnisse beschreiben pandemiebedingte Änderungen im Versorgungsalltag aus Sicht stationär tätiger Psychiater*innen sowie mögliche Auslöser von MD und beinhalten Bewertungen der Häufigkeit und Intensität ihres Auftretens. Die im Rahmen unserer Befragung benannten Auslöser deuten auf eine Vielzahl bisher noch wenig beleuchteter moralischer Herausforderungen hin, von denen einige im Hinblick auf zukünftiges Krisenmanagement und vor dem Hintergrund möglicher Unterstützungsangebote durch klinische Ethik diskutiert werden sollten.

Ein häufiger und starker Auslöser für MD war in dieser Studie die erschwerte Kommunikation durch den MNS. Dies unterstreicht die herausragende Bedeutung des Gesichtsausdruckes für die Gestaltung der Beziehung zwischen Ärzt*innen und Patient*innen insgesamt, aber auch für die Kommunikation im psychotherapeutischen Setting im Besonderen [24]. Es macht deutlich, dass die nonverbale Kommunikation für die Akteur*innen über handlungspraktische Konsequenzen hinaus auch wesentlich eine moralische Funktion haben kann. Die herausgehobene Bedeutung der Kommunikation zwischen Ärzt*innen und Patient*innen korrespondiert mit anderen Studien während der COVID-19-Pandemie, bei denen sich MD zeigt, wenn die Kommunikation durch Hygienemaßnahmen beeinträchtigt wird [11].

Eine hohe Relevanz wurde von den Teilnehmenden der erschwerten Begleitung von Patient*innen durch Angehörige aufgrund von Besuchseinschränkungen und -verboten zugeschrieben. Die Zugangsbeschränkungen zu Kliniken wurden bereits im Vorfeld als ein zentraler Konflikt der Pandemie mit moralischer Relevanz herausgearbeitet [6, 25–27]. Darüber hinaus wird der Bedarf eines an das jeweilige Infektionsgeschehen angepassten, ethisch reflektierten Besuchskonzeptes von Klinken deutlich, das auch gut begründete Einzelfallentscheidungen ermöglichen sollte [26].

Die qualitativen Ergebnisse deuten darauf hin, dass in manchen Kliniken ökonomische Interessen gegen Infektionsschutz abgewogen werden mussten. Bereits vor der Pandemie machte eine deutsche qualitative Interviewstudie von Entscheidungsträger*innen im Gesundheitswesen auf ethische Konflikte durch eine zunehmende Ökonomisierung von Gesundheitsangeboten aufmerksam, wobei betriebswirtschaftliche Ziele vornehmlich als hinderlich für die Erfüllung der adäquaten Patient*innenversorgung gesehen wurden. Als besonders negativ wurde hierbei die indirekte Einflussnahme von Geschäftsführungen auf die Personaldichte und Ressourcenverfügbarkeit bewertet, die dem eigentlichen Bedarf nicht entsprachen [28]. Eine wesentliche normative Anforderung ist dabei, dass Rahmenbedingungen bei begrenzten Ressourcen so gesteckt sein sollten, dass eine qualitativ hochwertige Versorgung gewährleistet werden kann und medizinische Entscheidungen nicht durch wirtschaftliche Faktoren fehlgeleitet werden [29].

Traditionell sind auch (fehlende) Entscheidungen im Zusammenhang mit einer Therapiezieländerung hin zu einem palliativen Therapieziel mit MD assoziiert [30, 31]. Ein palliatives *Psychiatriekonzept* wurde vorgeschlagen, um angemessen mit Patient*innen umgehen zu können, die auch trotz wiederholter Behandlungsversuche keine Behandlungsfortschritte erzielen [32]. Erwägungen im Zusammenhang mit diesem Denkansatz spielen in der Praxis der stationären Psychiatrie bisher offenbar keine Rolle, auch nicht im Kontext der Pandemie.

Angebote zur klinischen Ethikberatung bei der Unterstützung von Gesundheitspersonal im Umgang mit MD zu nutzen, wurde schon vor der COVID-19-Pandemie diskutiert [33]. Während Kolleg*innen in den USA wie Morley et al. die Potenziale spezifischer MD-Konsultationen hervorheben [34], machen Kritiker deutlich, dass MD auch ein Resultat klinischer Ethikberatung sein kann [35]. Gerade organisationsethische Angebote scheinen auch einen besonders positiven Effekt auf MD zu haben, was z. B. im Kontext von Entscheidungen über das Therapieziel bereits positiv hervorgehoben wurde [36]. Bei Entscheidungen auf der Organisationseben, die z. B. von Krisenstäben getroffen werden, sollte der MD von Mitarbeitenden berücksichtigt werden und im Einzelfall als auch in der Beratung auf Organisationsebene können Ethikberatungsangebote die Erarbeitung klinikinterner Regelungen unterstützen [26].

Die Ergebnisse der Befragung unterstreichen die Bedeutung von Unsicherheit während der Pandemie. Es ergibt sich Unsicherheit durch fehlende Evidenz und sich widersprechende oder häufig wechselnde Vorgaben, es besteht auch intrinsische Unsicherheit im moralischen Handeln. Für das Krisenmanagement lässt sich daraus die Wichtigkeit transparenter und möglichst abgestimmter Empfehlungen folgern. Für die klinische Ethik unterstreichen die Ergebnisse, dass eine Beschäftigung mit dem Thema Unsicherheit und Möglichkeiten, diese zu strukturieren und reduzieren, weiterhin als wichtiges Forschungs- und Tätigkeitsfeld weiter erschlossen werden sollte [37, 38].

In den Freitextantworten stellte sich dar, dass die Infektionsschutzmaßnahmen nicht hinreichend als den speziellen Erfordernissen der psychiatrischen Patient*innenversorgung angepasst wahrgenommen wurden. Hieraus ergibt sich eine wichtige praktische Implikation für die weitere bzw. zukünftige Pandemieplanung hinsichtlich der spezifischen Berücksichtigung der Bedürfnisse besonders vulnerabler Patient*innengruppen in der Psychiatrie, wie auch bereits von Kaufman et al. gefordert [39].

## Limitation

Durch das Auswählen einer Gelegenheitsstichprobe und die geringe Anzahl der Teilnehmenden liegt keine Repräsentativität der Ergebnisse für alle stationär tätigen Psychiater*innen vor. Verschiedene Einflüsse der Pandemie (Höhe der Infektionszahlen, Auftreten von Varianten, unterschiedliche Pandemievorgaben in den Bundesländern) könnten die Antworten der Teilnehmenden dynamisch beeinflusst haben, da sie in einem Zeitraum von mehreren Monaten in unterschiedlichen Bundesländern erhoben wurden. Aufgrund eines akuten Forschungsbedarfs in einer zeitlich hoch dynamischen Pandemie, wurde in dieser Untersuchung die Priorität auf eine teil-offene, zeitnahe Datenerhebung, anstatt auf die Entwicklung eines validierten Testinstruments gelegt.

## Fazit für die Praxis

- Die Ergebnisse geben einen ersten Einblick in die Wahrnehmung von Moral Distress (MD) in der stationären psychiatrischen Versorgung während der COVID-19-Pandemie.
- Spezifische ethische Herausforderungen und verschiedene Unterstützungs- und Anpassungsbedarfe werden diskutiert.
- Die Spezifika der Psychiatrie sollten in Entscheidungsprozesse von Krisenstäben auf den verschiedenen Ebenen des Gesundheitssystems berücksichtigt werden.
- Es ergeben sich wichtige Hinweise für Angebote der klinischen Ethikberatung, die über ethische Einzelfallberatungen hinaus auch organisationsethische Fragestellungen betreffen.
- Psychiater*innen (und Ethikberater*innen) sollten in ihrer Ausbildung auf MD vorbereitet werden.

## Supplementary Information




