# Supplementary material for: Changes in the routine work and moral distress in psychiatric care during the COVID-19 pandemic: A survey among physicians active in inpatient care in Germany
Source: Nervenarzt. 2023 Jul 5;94(9):827–34. [Article in German] doi: 10.1007/s00115-023-01499-z (PMC10499671; doi:10.1007/s00115-023-01499-z)
Supplement: Supplementary file 1 [file 115_2023_1499_MOESM1_ESM.odt]

| Tabelle 1 Soziodemographische Daten (Gesamt: n=141) |
| --- |
| Merkmal | Beschreibung | n (%) |
| Geschlecht | männlich | 59 (41,8) |
|  | weiblich | 81 (57,4) |
|  | divers | 0 (0) |
|  | keine Angabe | 1 (0,7) |
| Alter | 20-30 Jahre | 24 (17,0) |
|  | 31-40 Jahre | 38 (27,0) |
|  | 41-50 Jahre | 33 (23,4) |
|  | 51-60 Jahre | 34 (24,1) |
|  | älter als 60 Jahre | 11 (7,8) |
|  | keine Angabe | 1 (0,7) |
| Berufserfahrung | weniger als 1 Jahr | 4 (2,8) |
|  | 1-5 Jahre | 29 (20,6) |
|  | 6-10 Jahre | 27 (19,1) |
|  | 11-15 Jahre | 19 (13,5) |
|  | länger als 15 Jahre | 60 (42,6) |
|  | keine Angabe | 2 (1,4) |
| Position | Assistenzärzt*in | 58 (41,1) |
|  | Oberärzt*in | 50 (35,4) |
|  | Chefärzt*in | 22 (15,6) |
|  | andere | 6 (4,3) |
|  | keine Angabe | 5 (3,5) |
|  |

| Tabelle 2 |
| --- |
| Kategoriensystem zur Änderung des Arbeitsalltages |
| HK1 Auswirkung auf die Kommunikation im Arbeitsalltag |
| 1a. Änderung Kommunikationswege |
| HK2 negative Auswirkung auf die Kommunikation im Arbeitsalltag |
| 2a.Verschlechterung der Kommunikation im Team |
| 2b. Auswirkung der Hygienemaßnahmen auf die Kommunikation |
| 2c.Auswirkungen von Kontakteinschränkungen |
| 2d. Kommunikation von Regelungen |
| HK3 positive Auswirkung auf die Kommunikation im Arbeitsalltag |
| 3a. Verbesserung der Kommunikation im Team |
| HK4 positive Auswirkung auf die Organisation des Arbeitsalltages |
| 4a.Organisatorische Änderungen, die mehr Raum für die einzelnen Patient*innen ermöglichen |
| 4b. positive organisatorische Änderungen in Bezug auf Hygienemaßnahmen |
| HK5 negative Auswirkung auf die Organisation des Arbeitsalltages |
| 5a.Erhöhter Zeit- oder Planungsaufwand durch Organisationsmaßnahmen |
| 5b.Organisatorische Änderungen in Bezug auf Abläufe |
| 5c.Organisatorische Änderungen in Bezug auf Hygienemaßnahmen |
| 5d. Kommunikation mit den Patient*innen über die Umsetzung der Hygienemaßnahmen |
| HK6 Auswirkung auf die Arbeit mit Angehörigen |
| 6a.Angehörigenarbeit erschwert |
| HK7 Positive Auswirkung auf psychiatrische Behandlung |
| 7a.Therapieverbesserung durch geringere Patient*innenzahl |
| 7b.Bestmögliche Behandlung trotz Pandemie |
| HK8 Negative Auswirkung auf die psychiatrische Behandlung |
| 8a.Patient*innenversorgung eingeschränkt durch geringeres Therapieangebot |
| 8b.Negative Auswirkungen auf Therapeut/Patient*innenbeziehung |
| 8c.Behandlungsschwierigkeiten durch Hygienemaßnahmen |
| 8d.Leiden der Behandlungsqualität und Weiterbehandlung |
| 8e.Somatische im Vergleich zu therapeutischen Aspekten im Vordergrund |
| 8f.Beurteilbarkeit der psychischen Verfassung erschwert |
| 8g.Krankheitsverläufe, -schwere oder Art der Diagnosen der Patient*innen verändert |
| 8h.verschlechterte Sicht der Patient*innen auf die Therapie |
| 8i.Offenlegung von systemimmanenten Problemen |
| 8j.Regelhinweise als Teil des Patient*innenkontaktes notwendig |
| HK9 Auswirkung auf die Behandelnden |
| 9a.Soziale Interaktion Kolleg*innen erschwert/fehlend |
| 9b.Arbeitsplatzwechsel der Behandelnden durch Stationsschließungen |
| 9c.Infektionsgefahr für Behandelnde |
| 9d.Auswirkungen psychischer Art auf die Behandelnden |
| 9e.Personalausfall |
| 9f.Konfliktpotential zwischen Behandelnden bei der Behandlung infizierter Patient*innen |
| 9g.Einschränkungen in der Weiterbildung der Behandelnden |
| HK10 Veränderung der Mittelallokation |
| HK11 Moralischer Konflikt |
| 11a.Moralisch relevanter Konflikt bezüglich der Bettenplanung |
| HK12 Negative Gefühle |
| 12a.Ausgelöste Gefühle |
| HK13 Unsicherheit |
| 13a.Unsicherheiten im Umgang mit der Pandemie |
| HK14 Kritik an politischen Vorgaben zur Pandemieeindämmung |
| 14a.Verzögerte Reaktionen der Politik |
| HK15 Negative Auswirkung auf die autonome Lebensgestaltung von Patient*innen auf der Station |
| 15a. Einschränkung der Patient*innenautonomie im stationären Setting |
| 15b. Einschränkung des Soziallebens |
| HK16 Moralischer Konflikt zwischen dem individuellen Interesse der Patient*innen und dem wirtschaftlichen Interesse der Klinik |

| Tabelle 3 |
| --- |
| Kategoriensystem zur Sicht auf Empfehlungen und Maßnahmen |
| HK1 Gelungene Kommunikation und Umsetzung von Empfehlungen und Maßnahmen von Mesoebene |
| 1a. Empfehlungen/Maßnahmen von Klinikum/Führungsebene gegeben |
| HK2 Umgang mit Unklarheiten bezüglich der Pandemie |
| 2a. Verringerung der Unklarheiten im Verlauf |
| HK3 Unsicherheiten speziell für Pflegeteams (Mikroebene) |
| HK4 Unklarheiten in der Umsetzung von Empfehlungen durch häufige Änderungen |
| 4a. Unklarheiten durch ständige Änderungen |
| HK6 Problematische Umsetzung der Empfehlungen und Maßnahmen allgemein im klinischen Alltag |
| 6a. Fahrlässigkeiten im Umgang mit möglichen Infektionen |
| 6b. notwendige Kontrollen der Maßnahmen |
| 6c. Mängel in der Umsetzung der Empfehlungen und Maßnahmen |
| HK7 problematische Umsetzung der Empfehlungen und Maßnahmen speziell im psychiatrischen klinischen Alltag (Mikroebene) |
| 7a. nicht auf psychiatrische Behandlung angepasste Handlungsanweisungen (Mikroebene) |
| HK8 Problematische Umsetzung der Empfehlungen und Maßnahmen speziell im psychiatrischen klinischen Alltag (ohne Ebene) |
| 8a. nicht auf psychiatrische Behandlung angepasste Handlungsanweisungen (ohne Ebene) |
| HK9 Problematische Umsetzung der Empfehlungen und Maßnahmen speziell im psychiatrischen klinischen Alltag (Makroebene) |
| 9a. nicht auf psychiatrische Behandlung angepasste Handlungsanweisungen (Makroebene) |
| HK10 Ambivalente Bewertung der Empfehlungen und Maßnahmen |
| 10a. notwendige Weiterentwicklung der Empfehlungen und Maßnahmen im Verlauf für mehr Handlungsspielraum auf Mikroebene |
| 10b. Ambivalenz bezüglich der Empfehlungen und Maßnahmen |
| HK12 Gelungene Umsetzung der Empfehlungen und Maßnahmen auf Mikroebene |
| HK13 gelingende Kommunikation über Empfehlungen und Maßnahmen durch Bildung eines Krisenstabs |
| HK14 Moralischer Konflikt zwischen Patientenwohl und ökonomischen Interessen der Einrichtung |
| 14a. Wirtschaftlichkeit in Konkurrenz mit infektiologischer Sicherheit |
| HK15 Moralischer Konflikt zwischen individuellem Patientenwohl und Infektionsschutzempfehlungen (Public health) |
| 15a. Patient*innenversorgung in Konkurrenz mit Empfehlungen/Maßnahmen |
| HK16 Moralischer Konflikt: Gerechtigkeitsfrage zwischen verschiedenen Patient*innengruppen |
| HK17 Erschwernis der Durchsetzung von Kontaktbeschränkungen führt zu erhöhtem Infektionsrisiko für Mitarbeitende |
| HK18 Empfehlungen von Makroebene vermindern die Möglichkeiten von Empfehlungen auf Mesoebene |
| HK19 Negative Auswirkung auf Patient*innen |
| HK20 erschwerte Beziehungsaufnahme durch Maßnahmen/Empfehlungen |
| HK21 Kritik an Empfehlungen Makroebene |
| HK22 Hohe Bedeutung von Empfehlungen und Maßnahmen von Mesoebene |
| 22.a Empfehlungen/Maßnahmen von Klinikum/Führungsebene abhängig |
| HK23 Kritik an den Empfehlungen und Kommunikation dieser ohne Ebenenzuordnung |
| 23a. Widersprüchlichkeit/Unklarheit der Anweisungen allgemein |
| HK24 Positive Bewertung der Empfehlungen und Maßnahmen |
| 24a. Angemessenheit der Empfehlungen und Maßnahmen |
| HK25 Mangel an materiellen und personellen Ressourcen |
| HK26 Kommunikations- und Umsetzungsprobleme im Austausch mit dem Gesundheitsamt |
| 26a. Widersprüchlichkeit/Unklarheit der Anweisungen des Gesundheitsamtes |
| HK27 Negative Bewertungen der Empfehlungen ohne Zuordnung zur Ebene |
| HK28 Negative Bewertungen der Empfehlungen von der Makroebene |
| 28a. Unklarheiten durch politische Vorgaben |
| HK29 Negative Bewertung der Kommunikation von Infektionsschutzmaßnahmen |
| 29a. Unklarheiten durch Informationsmangel |
| HK30 Negative Auswirkungen für Menschen in Heimen und Werkstätten durch Kontaktbeschränkungen |
| HK31 Negative Bewertung der Empfehlungen und Maßnahmen ohne Ebenenzuordnung |
| 31a. Einschnitt in die Eigenverantwortlichkeit |
